# MRI texture analysis (MRTA) of T2-weighted images in Crohn’s disease may provide information on histological and MRI disease activity in patients undergoing ileal resection

**DOI:** 10.1007/s00330-016-4324-4

**Published:** 2016-04-05

**Authors:** Jesica Makanyanga, Balaji Ganeshan, Manuel Rodriguez-Justo, Gauraang Bhatnagar, Ashley Groves, Steve Halligan, Ken Miles, Stuart A. Taylor

**Affiliations:** 1 Center for Medical Imaging, University College London and University College London Hospitals NIHR Biomedical Research Centre, 250 Euston Road, London, NW1 2BU UK; 2 Institute of Nuclear Medicine, University College London, University College Hospital, 235 Euston Road, London, NW1 2BU UK; 3 Department of Research Pathology, University College London Cancer Institute, University College London, Rockefeller Building, London, WC1E 6JJ UK

**Keywords:** Crohn disease, Magnetic resonance imaging, Textural analysis, Small intestine, Histology

## Abstract

**Objectives:**

To associate MRI textural analysis (MRTA) with MRI and histological Crohn’s disease (CD) activity.

**Methods:**

Sixteen patients (mean age 39.5 years, 9 male) undergoing MR enterography before ileal resection were retrospectively analysed. Thirty-six small (≤3 mm) ROIs were placed on T2-weighted images and location-matched histological acute inflammatory scores (AIS) measured. MRI activity (mural thickness, T2 signal, T1 enhancement) (CDA) was scored in large ROIs. MRTA features (mean, standard deviation, mean of positive pixels (MPP), entropy, kurtosis, skewness) were extracted using a filtration histogram technique. Spatial scale filtration (SSF) ranged from 2 to 5 mm. Regression (linear/logistic) tested associations between MRTA and AIS (small ROIs), and CDA/constituent parameters (large ROIs).

**Results:**

Skewness (SSF = 2 mm) was associated with AIS [regression coefficient (rc) 4.27, *p* = 0.02]. Of 120 large ROI analyses (for each MRI, MRTA feature and SSF), 15 were significant. Entropy (SSF = 2, 3 mm) and kurtosis (SSF = 3 mm) were associated with CDA (rc 0.9, 1.0, −0.45, *p* = 0.006–0.01). Entropy and mean (SSF = 2–4 mm) were associated with T2 signal [odds ratio (OR) 2.32–3.16, *p* = 0.02–0.004], [OR 1.22–1.28, *p* = 0.03–0.04]. MPP (SSF = 2 mm) was associated with mural thickness (OR 0.91, *p* = 0.04). Kurtosis (SSF = 3 mm), standard deviation (SSF = 5 mm) were associated with decreased T1 enhancement (OR 0.59, 0.42, *p* = 0.004, 0.007).

**Conclusions:**

MRTA features may be associated with CD activity.

***Key Points*:**

• *MR texture analysis features may be associated with Crohn’s disease histological activity*.

• *Texture analysis features may correlate with MR-dependent Crohn’s disease activity scores*.

• *The utility of MR texture analysis in Crohn’s disease merits further investigation*.

**Electronic supplementary material:**

The online version of this article (doi:10.1007/s00330-016-4324-4) contains supplementary material, which is available to authorized users.

## Introduction

MR enterography is established for assessment of disease activity in small bowel and colonic Crohn’s disease. Mural features such as thickness [1–3], T2 signal intensity [3, 4], and contrast enhancement [1–4] are significantly correlated with endoscopic and histological disease activity and MRI is used routinely to assesses global disease burden [5] and monitor treatment response [6].

Texture analysis (TA) is a novel image analysis technique that can quantify image heterogeneity resulting from changes not appreciated by the human eye [7, 8]. Disease processes may introduce image heterogeneity and TA has been studied in many cancers including lung [9], breast [10], and colorectal [11]. The underlying rationale is that complex microscopic tumour heterogeneity resulting in structures of different sizes and variation may be reflected indirectly by the distribution of greyscale levels and/or pixel intensity on diagnostic images such as CT and MRI (i.e. macroscopic heterogeneity). In cancer, changes in image texture are associated with hypoxia, angiogenesis, cellular proliferation, tumour grade, genetic mutation status [12, 13], and with prognosis and treatment response [14, 15].

The hallmark of active Crohn’s disease on histology includes transmural inflammation, fissuring ulcers, submucosal expansion/oedema, and inflammation-driven new vessel formation [16]. It is plausible that these may be reflected by texture changes so that texture analysis could refine the use of MRI as a biomarker of disease activity further.

One sequence ubiquitous to MR enterography protocols is T2-weighted imaging. Radio-pathological comparisons using surgical specimens have shown significant linear correlations between mural T2 signal and inflammatory activity [4, 17, 18], and assessment of T2 signal is common to MRI activity scores such as the MaRIA [1] and Crohn’s disease activity scores [19].

The aim of our study was to explore whether MRTA measured in T2-weighted MRI images is associated with histological and MRI scores of disease activity.

## Materials and methods

### Study population

The institutional review board issued a waiver for re-consenting patients for additional data analysis. A retrospective analysis was undertaken using data collated between July 2006 and December 2007 from 18 (mean age 31 years, 9 male) consecutive patients with proven Crohn’s disease (based on standard clinical, endoscopic, and histological criteria) undergoing MR enterography and scheduled to undergo elective small bowel resection for disease-related complications [17].

The original study aimed to validate proposed MR enterography features of Crohn’s disease activity using a transmural histopathological reference standard. The current study used the collated MRI data sets and same histopathological reference standard and additionally applied MRTA to axial T2-weighted images. Two patients from the original study were excluded because axial T2-weighted images were unavailable for analysis because movement artefact degraded the image quality.

The Montreal classification was recorded for each patient. Harvey Bradshaw index and C-reactive protein were also recorded within the 5 days preceding surgery (Table 1).

**Table 1:** Demographic characteristics of included patients

Age	Montreal classification	Harvey Bradshaw index	CRP (mg/L)
17	A1L3B1	7	27
16	A1L3B3	5	157
21	A2L3B2	8	7
26	A2L3B2	4	<5
28	A2L3B1	4	84
16	A1L3B3p	8	30
40	A1L3B2	17	107
51	A2L3B2	6	113
40	A2L3B2	4	70
27	A1L3B1	16	66
49	A3L3B1	7	142
19	A1L3B2	4	19
56	A3L3B1	4	184
47	A3L1B1	15	32
16	A2L3B1	6	70
29	A2L3B1	10	114

### MR enterography protocol

Full details of the MR enterography protocol and subsequent matching of sites of histological sampling to preoperative MR enterography were as described previously [17]. Patients underwent MR enterography within 2 weeks of surgery (mean 4 days, range 1–13 days) on a 1.5-T MRI unit (Avanto; Siemens, Erlangen, Germany)

As part of the standard MR enterography protocol, axial single shot turbo spin echo (SSTSE) sequences were obtained (TR 800 ms, TE 86 ms, matrix 256 × 195) together with pre- and post-contrast T1 VIBE images (Table 2)

**Table 2:** MR imaging parameters for preoperative patient imaging and post-resection specimen imaging

	Clinical scan				Specimen scan
Parameter	Coronal and axial half-Fourier RARE sequence	Coronal and axial True FISP sequence	Baseline VIBE sequence	30-s and 70-s post contrast VIBE sequence	Half-Fourier RARE sequence
Field of view (mm)	Variable	Variable	Variable	Variable	Variable
No. of sections	20/26	25/34	48	48	15
No. of stacks	1/4	1/2	1	1	1
Repetition time (ms)	1200/800	4/4.2	7.2	7.2	1200
Echo time (ms)	86/86	1.7/2.1	2.4	2.4	84
Image matrix	256 × 195	256 × 205	256 × 135	256 × 135	256 × 195
Section thickness (mm)	4/4	4/4	3	3	2
Section gap (mm)	5.2/4.2	5.2/5.4	0	0	0
No. of measures acquired	1	1	1	1	1
Turbo factor	195	1	1	1	195
Integrated parallel acquisition technique	GRAPPA factor of 2	NA	NA	NA	GRAPPA factor of 2
Flip angle (°)	50	46	10	10	150

Within 24 h of surgery, the resected specimen was pinned to a board in its anatomical orientation and scanned using an SSTSE sequence in axial and coronal planes.

The study coordinator, in collaboration with the operating surgeon, reviewed the preoperative MR enterography to locate the exact segment of bowel resected (using fixed anatomical landmarks such as the ileo-caecal valve (ICV), site of fistula etc.) and then chose one to five sampling sites (median, three) through the resected bowel on the preoperative MRI scan for detailed histological correlation.

The coordinator in consensus with the study histopathologist (the latter with 15 years of experience in gastrointestinal histopathology) then located the selected sampling sites on the resected specimens. Sampling sites were co-located between MRI scan and the specimen using distance measurements with reference to fixed anatomical structures such as the ICV, fistula site, abscess etc.

### MRI Crohn’s disease activity (CDA) scoring

The preoperative MR enterography images were uploaded onto a standard picture archiving and communication system workstation (Agfa Healthcare UK, Brentwood, England). Segmental disease activity was scored in consensus by two gastrointestinal radiologists with 10 and 15 years’ experience respectively of MR enterography using the MR Crohn’s disease activity (CDA) score [19], previously validated against a histological reference standard. Specifically, activity in the region of all sampling sites in the subsequently resected segment was scored on a scale 0 to 3 for mural thickness, mural T2 signal, perimural T2 signal, and qualitative mural enhancement (maximum score = 12) [19] (see electronic supplementary material). The individual scores were summed to give the CDA score for each site. If a particular sampling site had a range of activity based on CDA scoring in the vicinity, the area attracting the highest CDA score was used for subsequent textural analysis.

### Region of interest placement and MR textural analysis

Axial T2-weighted images were uploaded into proprietary software for textural analysis (TexRAD, www.texrad.com, part of Feedback Plc, Cambridge, UK) [7].

Two regions of interest (ROI) types (“large” and “small”) were drawn freehand by two observers in consensus (a radiologist with 15 years of experience of MRI enterography and a research fellow with 2 years’ experience of MRI enterography). Observers were blinded to any histological analysis (other than the exact site of sectioning) but had full access to the complete MRI data sets including coronal T2- and T1-weighted images.

**Fig. 1:**
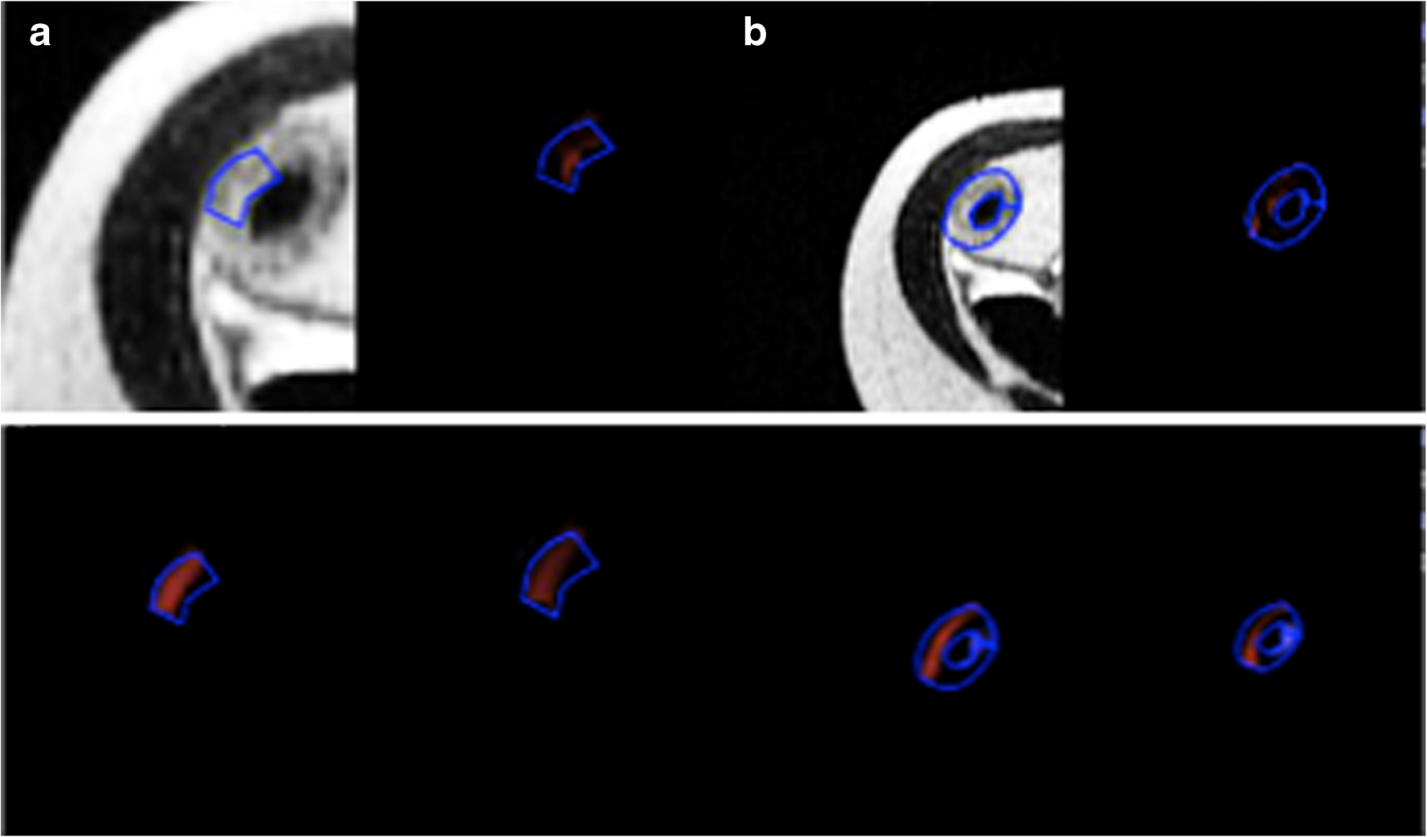
Examples of small (**a**) and large (**b**) regions of interest drawn on axial half-Fourier RARE sequence images in diseased bowel subsequently resected along with the texture maps at fine (*upper right panel*), medium (*lower left panel*), and coarse (*lower right panel*) texture scales. Small ROI = 42 pixels. Large ROI = 169 pixels

The observers drew a small ROI in each of the sampling sites previously identified for detailed histopathogical matching as described above. All available MRI sequences and orientations were used to locate these designated sites on the axial T2-weighted image. A small ROI was placed at each site. Observers were instructed to include the full bowel wall thickness within the ROI but to limit its width to 3 mm so as to match the exact site of histological sampling as closely as possible (Fig. 1a).

The observers then scrolled through the axial T2-weighted sequence and selected the slice which had been used to grade the MRI CDA as described above. A large ROI was drawn to include as much of the diseased segment as possible in this single slice. Care was taken to exclude any intraluminal or mesenteric tissue (Fig. 1b).

There were a total of 36 small ROIs and 36 large ROIs across the 16-patient cohort.

Finally, a further ROI was placed in the cerebrospinal fluid (CSF) in the same slice for normalization of textural parameters on the T2 weighted image (see below).

### Texture analysis

Textural analysis was performed for both ROI types using methodology previously described [10] and employing a filtration-histogram technique. Filtration extracts and enhances texture features at different sizes within ROIs and is followed by histogram quantification. MRTA was carried out using commercially available research software (TexRAD – www.texrad.com, Feedback Plc, Cambridge, UK) [7]. Specifically the initial filtration step employs a Laplacian of Gaussian (LoG) bandpass filtration, which extracts and highlights image features of different sizes corresponding to the spatial scale of the filter (SSF), ranging between 2 mm (fine textural features) and 6 mm (coarse features) in width (radius). Quantification of histograms (with and without filtration) was based on mean (average value of the pixels within the ROI), standard deviation (width of the histogram or degree of variation/dispersion from the average), skewness (symmetry of the distribution), mean of positive pixels (MPP, average of the pixel values which are positive), kurtosis (pointiness of the distribution), and entropy (with higher values indicating increasing image irregularity or complexity). A summary of the premise underlying the interpretation of TA parameters is as follows. The mean changes approximately in proportion to the number of objects highlighted and their mean brightness (dark objects are negative). The SD increases approximately in proportion to the square root of the number of objects highlighted by the filter and their mean intensity difference compared to background tissue (i.e. dark and bright objects are both positive); generally a higher SD value implies increased heterogeneity.

Skewness reflects the average brightness of highlighted objects (predominantly bright objects give positive skewness values and predominantly dark objects give negative skewness values). Skewness tends to zero with increasing number of objects highlighted and moves away from zero with intensity variations in highlighted objects.

Kurtosis is inversely related to the number of objects highlighted (whether bright or dark) and is increased by intensity variations in highlighted objects.

Because the gain factor for T2-weighted image acquisition can change between patients, the mean, SD, and MPP texture parameters (parameters potentially affected by this variation in gain factor) were normalized by dividing by the signal intensity of the CSF for each patient and at each filter SSF value.

### Histopathological assessment

Histological analysis was performed by a specialist gastrointestinal pathologist (with 15 years of experience, blinded to other information). Sections were stained with haematoxylin–eosin (H&E). At the specific sampling sites previously matched between the preoperative MRI and resected specimen (and corresponding to the placement of small ROI), acute inflammation was assessed on the basis of the method of Borley et al. [20] and an single acute inflammatory score (AIS) was calculated (electronic supplementary material).

### Statistical analysis

The primary analysis was to examine the association between each textural parameter and histological score of activity (AIS) for the matched small ROI.

Secondary analyses examined the association between textural parameters and the MRI activity score (and its mural components) using the large ROI.

Separate analyses were performed for each filter. Given the limited size of the small ROI, only SSF = 0, 2, and 3 mm were examined. For the large ROI analysis all filters (0, 2, 3, 4, and 5 mm) were applied.

All analyses used regression methods. Ordered logistic regression was used for the ordinal outcomes. AIS and total MRI score were both normally distributed and linear regression was used for these continuous outcomes. To account for the range of data for each textural parameter, odds ratios were expressed as follows: 50-unit increase (mean, SD, MPP), 1-unit increase (skewness, entropy, and kurtosis), and 10-unit increase (MRI CDA score). Fischer’s exact test was used to compare paired proportions as appropriate.

Within each patient there was more than one ROI analysed, i.e. there were multiple measurements per patient that therefore were non-independent. Robust standard errors (Huber White) were used to account for this. The level of significance was defined as *p* < 0.05 for all analyses.

## Results

Mean patient age was 39.5 years (range 16–66 years). Full demographic characteristics are in Table 1.

The range of MRTA parameters according to ROI size is shown in Table 3.

**Table 3:** Mean and range of values for each MR textural analysis parameter for small and large ROIs

SSF	Mean (range)	SD (range)	Entropy (range)	MPP (range)	Skewness (range)	Kurtosis (range)
Small ROI						
0	389.6 (195.4–580.3)	119.4 (52.4–343.9)	3.7 (2.9–4.4)	389.6 (195.4–580.3)	1.0 (−0.2 to 2.8)	1.2 (−1.3 to 12.1)
2	−142.7 (−582.5 to 269)	146.3 (0–467.5)	1.8 (0–3.5)	89.2 (0–269)	0.1 (−0.4 to 0.8)	−1.0 (−2.0 to −0.3)
3	−189.9 (−688.5 to 134.2)	82.5 (0–214)	1.6 (0–3.4)	0 (0–178.2)	0 (−0.8 to 1.2)	−1.0 (−2 to 0.0)
Large ROI						
0	400.5 (217.3–548.5)	135.5 (49.7–324.3)	4.9 (4.1–5.3)	400.5 (217.3–548.5)	1.2 (−0.8 to 2.7)	2.2 (−1.1 to 9.4)
2	−121.5 (−612.5 to 128.2)	185.9 (0–793.7)	3.9 (0–5.2)	125.4 (0–1559.5)	0.1 (−1.0 to 1.9)	−0.0 (−1.0 to 7.0)
3	−225.3 (−549.5 to 45.6)	161.9 (0–850.4)	3.6 (0–5.2)	97.2 (0–1190.5)	0.2 (−0.8 to 3.1)	−0.3 (−1.4 to 10)
4	−189.1 (−509 to 2.9)	91.6 (0–415.1)	2.4 (0–4.9)	35.1 (0–438.2)	0 (−1.2 to 1.1)	−0.5 (−1.7 to 0.4)
5	−160.4 (−471.5 to 11.8)	74.6 (0–279.9)	1.8 (0–4.9)	0 (0–293.5)	0.2 (−1.2 to 1.2)	−0.7 (−1.2 to 0.9)

### MRTA and histological activity scores (small ROI)

The mean size of the small ROI was 52 pixels, SD 23.5 (range 19–118).

The mean AIS was 4.53, SD 3.6 (range 0–11).

**Fig. 2:**
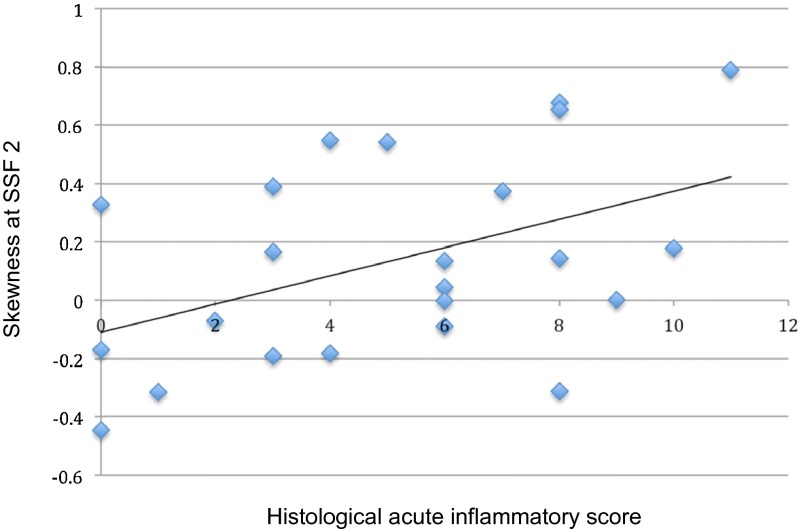
Scatter plot and line of regression between histological acute inflammation score (AIS) in the bowel wall and skewness at SSF 2 for the small ROI

There was a significant positive correlation between skewness at SSF = 2 mm and histological AIS [regression coefficient 4.27 (95 % CI 0.74, 7.79), *p* = 0.02] (Fig. 2).

**Fig. 3:**
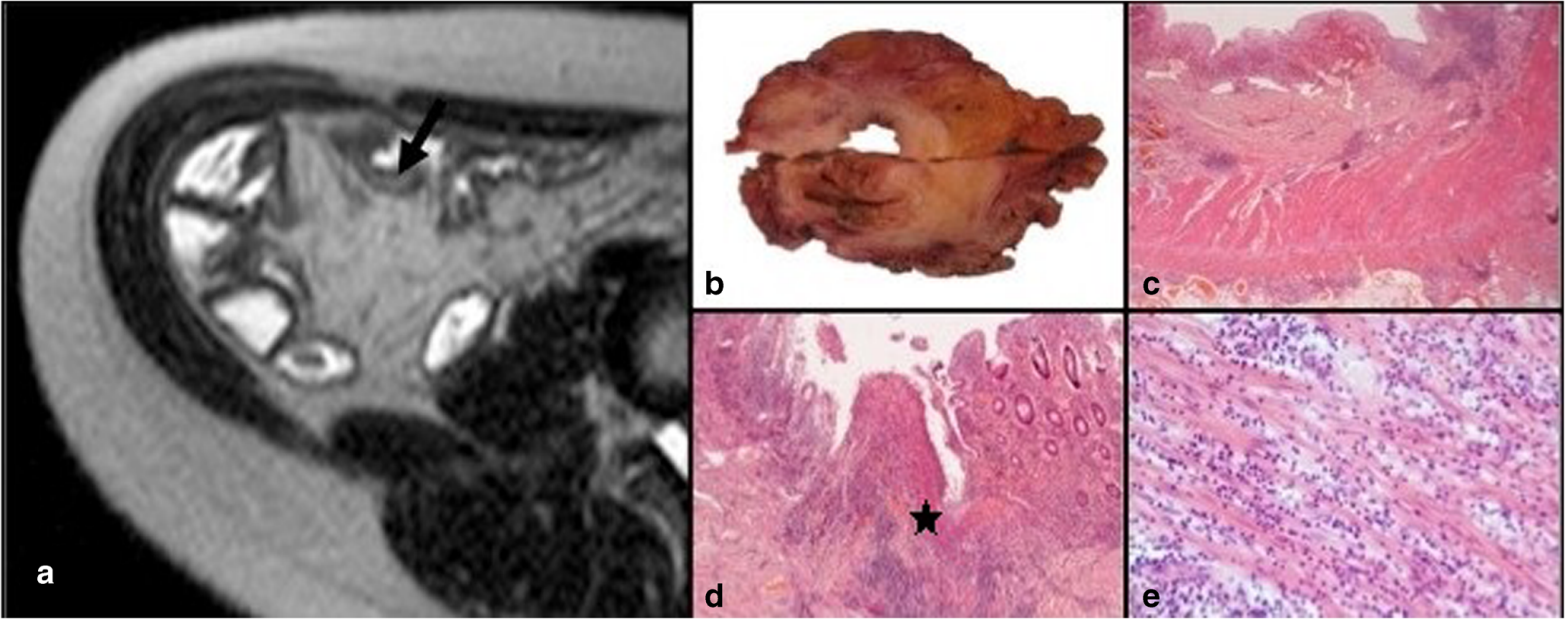
Anastomotic ileal recurrent Crohn’s disease with high skewness on MRTA and increased histological inflammation (AIS score of 7). **a** Axial T2-weighted coronal SSTSE through the neoterminal ileum (*arrow*) attracting a Crohn’s disease activity score of 9 (large ROI) and skewness of 0.5346 (small ROI). **b** Coronal section of the bowel through the resection specimen matched to the site of sampling on MRI. **c** Full thickness H&E stained histological section (×12.5 magnification) showing mucosal ulceration and transmural inflammation with lymphoid aggregates. **d** H&E-stained histological section, ×100 magnification) shows confluent mucosal ulceration. **e** H&E-stained histological section (×200 magnification) taken from the region of the *star* in D shows an acute inflammatory cell infiltrate extending into the muscularis propria

At SSF = 2 mm, 10 of 23 small ROIs had an AIS ≤ 4 of which 4 (40 %) exhibited skewness ≥ 0. Conversely 13 small ROIs had an AIS > 4 of which 11 (85 %) exhibited skewness of ≥0 (*p* = 0.04) (Fig. 3).

There were no other significant associations between textural parameters and AIS (*p* = 0.261–0.418).

### MRTA and MRI activity score (large ROI)

The mean size of the large ROI was 238 pixels, SD 107.7 (range 90–454).

The mean MRI CDA score was 8.8, SD 3.6 (range 3–14). A summary of the significant associations between texture parameters and mural MRI features according to SSF is shown in Table 4. For the large ROI, 15 of 120 associations (4 MRI scores, 6 MRTA parameters, and 5 filter levels) analysed were significant.

**Table 4:** Summary of significant associations between texture features and mural MRI features for the large ROI

Filter size (mm)	MRI feature	Textural parameter	Odds ratio (95 % CI)	*P* value
2	Mural thickness	MPP^a^	0.91 (0.83, 1.00)	0.04
2	Mural T2 signal	Mean^a^	1.22 (1.02, 1.47)	0.03
		Entropy^b^	3.16 (1.44, 6.95)	0.004
2	Mural enhancement	MPP^a^	0.91 (0.86, 0.97)	0.003
3	Mural T2 signal	Mean^a^	1.26 (1.01, 1.58)	0.04
		Entropy^b^	2.76 (1.20, 6.37)	0.02
3	Mural enhancement	MPP^a^	0.86 (0.75, 0.99)	0.04
		Kurtosis^b^	0.59 (0.41, 0.84)	0.004
4	Mural T2 signal	Mean^a^	1.28 (1.02, 1.61)	0.03
		Entropy^b^	2.32 (1.12, 4.83)	0.02
5	Mural enhancement	SD^a^	0.42 (0.22, 0.78)	0.007
		MPP^a^	0.18 (0.07, 0.48)	0.001

^a^Odds ratio reported for a 50-unit increase in predictor variable

^b^Odds ratio reported for a 1-unit increase in predictor variable

**Fig. 4:**
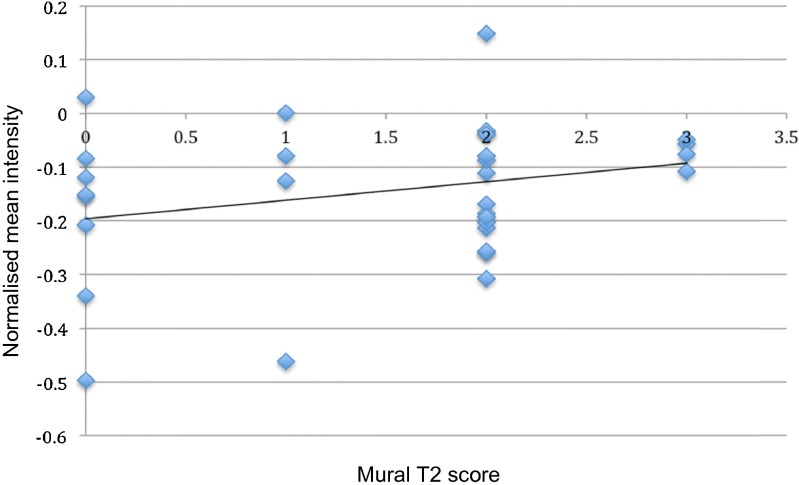
Scatter plot and line of regression between normalised mean intensity and T2 MRI score (subjectively graded from 0 to 3 depending on intensity of mural T2 signal) at SSF 2 for the large ROI

There were significant positive correlations between mean intensity values at different filter values (SSF 2, 3, and 4 mm) and T2 signal score [greatest significance at SSF = 2 mm, odds ratio (OR) 1.22 (1.02–1.47), *p* = 0.03] (Fig. 4, Table 4).

There were also associations between mural enhancement grade and MPP, kurtosis, and SD at several filter levels (Table 4). MPP in particular was significantly associated at filter values SSF 2, 3, and 5 mm, [OR 0.91 (0.86–0.97), *p* = 0.003; OR 0.86 (0.75–0.99), *p* = 0.04; and OR 0.18 (0.07–0.48), *p* = 0.001, respectively]. In general the enhancement grade tended to decrease for a rise in MPP.

There was a significant correlation between normalized MPP (SSF = 2 mm) and decreasing mural thickness [OR 0.91 (95 % CI 0.83, 1.00), *p* = 0.04].

Mean and entropy were positively associated with mural T2 signal. Entropy in particular was correlated with mural T2 signal intensity at SSF 2, 3, and 4 mm (OR 2.32–3.16, *p* = 0.02–0.004).

A significant correlation was demonstrated between total MRI CDA score and entropy (SSF = 2 mm and 3 mm), regression coefficient 1.00 (0.34, 1.65), and 0.90 (0.22, 1.58) (*p* = 0.006, *p* = 0.01), respectively. Kurtosis (SSF 3 mm) was negatively associated with MRI CDA, regression coefficient −0.45 (−0.70, −0.19) (*p* = 0.002).

## Discussion

Based mainly on applications in cancer imaging, it is known that MRTA can reflect underlying histological phenotypes. Data from the present study suggests that MRTA may also reflect inflammatory phenotype in Crohn’s disease.

Following careful matching of ROI size and positioning with the site of histological sampling, we found a significant positive correlation between pixel skewness and the histological acute inflammatory score at filter level 2 mm. In support, the number of small ROIs at SSF = 2 mm with skewness ≥0 was significantly greater for those with an AIS exceeding 4, compared to those with less active disease (AIS lower than 4). Filtered skewness provides pixel distributional information on the filtered image and skewness ≥0 reflects a preponderance of bright objects in the original MRI T2 image. It is therefore interesting to speculate as to what this relationship reflects. In T2-weighted MR images, the signal intensity is derived mainly from intracellular and extravascular extracellular space. Crohn’s disease activity both at endoscopy and histology is associated with higher T2 signal intensity, likely in part reflecting acute inflammatory infiltration and oedema [16], but given this we may have expected unfiltered texture parameters (skewness, mean intensity value, and MPP) to be significantly associated with the histological activity score. It seems likely the association between histological activity and simple unfiltered texture parameters on an T2 weighted image is therefore an overly simplistic way of assessing activity. Fine (2 mm) filtered texture features (skewness) are perhaps more sensitive and likely to reflect the subtle bright (e.g. water and fat) and dark (e.g. fibrosis) features linked to underlying histological phenotype. Skewness has been associated with angiogenesis in several neoplasms [7, 9, 21]. Furthermore, in the large ROI there was an association between mural enhancement and MPP on T2 weighted images, supporting the concept that T2 signal is influenced by tissue vascularity in CD. By using T2-weighted images rather than contrast-enhanced sequences we are of course limited with regard to the amount of information we can infer regarding inflammation-driven neoangiogenesis (which is well described in Crohn’s disease [22]). Nevertheless, pixel distributions in a CD segment on T2-weighted images will be influenced by many parameters including inflammatory cellular infiltration, cellular and interstitial oedema, and blood vessel density and distribution.

Although most work in textural analysis has concentrated on neoplasia and used CT, recent data also suggests textural changes on MRI reflect inflammatory damage in brain, skeletal muscle, and cartilage [23–25]. Indeed, Mahapatra et al. recently reported the use of textural features such as skewness and kurtosis on post-contrast-enhanced T1-weighted images to semi-automatically locate bowel afflicted by Crohn’s disease [26]

Although use of the large ROI and comparison with an MRI CDA is inferior to detailed histopathogical correlation, such an approach can provide a noninvasive insight regarding interpretation and potential utility of TA in MR enterography. Furthermore, MRTA may have a role by providing a more objective measurement of signal values rather than subjective grading by radiologists, who are known to suffer interobserver variation [27].

Entropy is a measure of signal irregularity within the ROI and was positively associated with T2 signal at three filter levels. Entropy was also positively associated with the MRI CDA score (although this does include a score of T2 signal, so the two observations are not independent). “Chaotic” and complex histology in active CD (reflected by image heterogeneity or complexity) would contrast with the more uniform appearances of chronic fibrotic disease, which would intuitively provide a link between signal entropy and activity. It is, however, notable that we found no such association using the small ROI matched with histology and so this association remains speculative.

The inverse correlation between total MRI CDA score and kurtosis is also interesting. Kurtosis is inversely related to the number of objects highlighted (whether bright or dark) and we can hypothesize that a greater number of highlighted objects reflects the more chaotic histology seen in active disease.

The total MRI score includes scores for contrast-enhanced sequences as well as extra mural signs such as perimural T2 signal which may influence how the total MRI activity score correlates with textural analysis in the T2-weighted sequence alone. Clearly, TA of contrast-enhanced T1-weighted images should be performed going forward. This was unfortunately not possible as the original MR enterography protocol utilised T1-weighted images acquired in the coronal plane only with non-isotropic voxels, impeding accurate TA. Another weakness was that we did not use fat-saturated T2-weighted images which were also not consistently available. It would be interesting to speculate if the data would be different if the fat signal from the bowel wall was nullified first, as this would perhaps have strengthened the association between TA parameters and histological inflammation.

Our study has other limitations. The sample size is small although reasonable for exploratory histopathological imaging correlative research of a relatively novel technology. Using a patient cohort undergoing surgical resection risks spectrum bias since such patients will have more advanced disease than those newly diagnosed. However this is unavoidable if full-thickness histopathological correlates are required, but the results are only applicable to this patient cohort. Bowel wall is a relatively small structure compared to larger tumours where TA has been applied successfully and repeatedly. We were careful to restrict ROIs to the bowel wall, but bowel lumen or mesentery may have been included inadvertently. Of note, TA has been applied to very small structures such as knee cartilage and, by definition, the bowel is usually thickened significantly in Crohn’s disease. The filtration-histogram TA approach is a key step towards extracting and quantifying texture features selectively at different filter sizes (SSF), related to tissue biology. Filtration at a scale above 2 mm minimizes the impact of image noise (more impacted at scales less than 2 mm) and further normalization of relevant texture quantifiers (minimizing the impact of variation in MR acquisition parameters) makes our approach more robust. Nevertheless, the restricted size of bowel ROI limits the volume of texture data that can be derived, and for this reason we limited the range of filters used.

Whilst the MRI CDA is validated as a score of activity, evaluation of T2-weighted images is an important part of the score and so MRTA metrics based on T2-weighted images may perhaps be expected to correlate with the CDA. Whilst we attempted to normalize T2 signal to CSF, it would likely have been better to normalise the whole image as the relationship between texture values and initial image brightness, strictly speaking, is nonlinear. However other workers have successfully used MRTA in non-normalised T2-weighted images in breast cancer [28], so we feel our approach was reasonable.

A multivariate statistical analysis would be more meaningful in future studies with a larger sample size.

It is interesting to speculate how MRTA could be used in clinical practice. The underlying premise is that macroscopic imaging features are a marker for microscopic histological phenotype. Most work has been done in cancer, where TA parameters are associated with genetic mutation status, hypoxia and angiogenesis, and even long-term prognosis. The utility of TA on patient management, however, remains somewhat controversial with unresolved questions regarding reproducibly and robustness of the methods (reflected in part by the variability in the data of the current study). Perhaps the most likely role of MRTA in Crohn’s disease would be as an objective marker of treatment response, where a measurable change in skewness for example acts as a biomarker for reduced histological inflammation. A logical next step would be to investigate MRTA before and after treatment.

Clearly with such a small sample, our conclusions are speculative at this stage, and a larger sample size may have produced a wide range of TA parameter correlation to histopathological activity grading.

The use of multiple regression analysis may produce spurious or chance associations. Just 15/120 large ROI correlations were indeed significant. Thus, our data must be viewed critically but merits future investigation.

## Conclusion

Our preliminary data suggests that some MRTA parameters may be associated with histological and MRI activity scores. Skewness measured using MRTA at a 2-mm filter level is potentially associated with a histological CD activity score. Additional MRTA features including kurtosis and entropy may also be associated with a validated MRI activity score (Crohn’s disease activity score). Such associations now need to be confirmed with larger sample sizes and appropriate statistical modelling to assess whether MRTA can act as an imaging biomarker of Crohn’s disease actively.

## Electronic supplementary material

Below is the link to the electronic supplementary material.

ESM 1 (DOCX 19 kb)

## Abbreviations

CDAS: Crohn’s disease activity score
LoG: Laplacian of Gaussian
MaRIA: Magnetic resonance index of activity
MRTA: MRI textural analysis
SSF: Spatial scale of the filter
