# Supplementary material for: MRI texture analysis (MRTA) of T2-weighted images in Crohn’s disease may provide information on histological and MRI disease activity in patients undergoing ileal resection
Source: Eur Radiol. 2016 Apr 5;27(2):589–97. doi: 10.1007/s00330-016-4324-4 (PMC5209452; doi:10.1007/s00330-016-4324-4)
Supplement: Supplementary file 1 — (DOCX 19 kb) [file 330_2016_4324_MOESM1_ESM.docx]

**On line appendix**

**1. MRI Scoring of Crohn’s disease activity**

| Score | 0 | 1 | 2 | 3 |
| --- | --- | --- | --- | --- |
| Mural thickness^a^ | 1–3 mm | >3–5 mm | >5–7 mm | >7 mm |
| Mural T2 signal^b^ | Equivalent to normal bowel wall | Minor increase in signal-bowel wall appears dark grey on fat saturated images | Moderate increase in signal-bowel wall appears light grey on fat saturated images | Marked increase in signal-bowel wall contains areas of white high signal approaching that of luminal content |
| Perimural T2 signal | Equivalent to normal mesentery | Increase in mesenteric signal but no fluid | Small fluid rim (≤2 mm) | Larger fluid rim (>2 mm) |
| Enhancement^c^ | Equivalent to normal bowel wall | Minor enhancement – bowel wall signal greater than normal small bowel but significantly less than nearby vascular structures | Moderate enhancement – bowel wall signal increased but somewhat less than nearby vascular structures | Marked enhancement – bowel wall signal approaches that of nearby vascular structures |

^a^ Measured using electronic calipers.

^b^ Compared to normal small bowel.

^c^ Compared to nearest vessel.

**3. Histological Acute Inflammatory Score (AIS) (based on Borley et al [21]**

| Mucosal ulceration | 0 – None  1 – Apthous ulcer (<7 mm)  2 – Linear ulcer  3 – Confluent-large ulcer |
| --- | --- |
| Oedema | 0 – None  1 – Mild  2 –Moderate  3 – Severe |
| Neutrophils | 0 – No increase  1 – Mild increase  2 –Moderate increase  3 – Marked increase |
| Depth of neutrophilic penetration | 0 – None  1 –Mucosa  2 – Submucosa  3 – Muscularis  4 – Serosa/extramural fat |
